# Disclosing the use of donor gametes to donor-conceived children: best practices to support the family unit

**DOI:** 10.1016/j.xfre.2026.03.004

**Published:** 2026-04-30

**Authors:** Sigal Klipstein, Lauri A. Pasch

**Affiliations:** a InVia Fertility Specialists, Chicago, Illinois; b Department of Psychiatry and Behavioral Sciences, University of California, San Francisco, California

**Case presentation**

A 52-year-old G1P1001 and her 57-year-old partner previously conceived their daughter using intrauterine insemination with anonymous donor sperm due to nonobstructive azoospermia. Their daughter “Abra” is now eighteen years of age and preparing to attend a local college. Abra’s father had a difficult time deciding to pursue this reproductive option and felt strongly that their use of donor sperm should not be shared with anyone, including their child. The mother asserts that this secret is not hers to keep and feels strongly that she should disclose the circumstances of Abra’s conception to her before she leaves for college. Abra’s mother has done some internet sleuthing and determined that the sperm donor’s identity is not so anonymous, and that he has fathered at least a dozen children. The family lives in a fairly small town, and the mother worries that her daughter may inadvertently and unknowingly meet—and perhaps even date—one of her half-siblings. The parents are struggling to determine if, when, and how to share the use of donor conception with Abra. They were able to reconsult with their original reproductive endocrinologist, who explained the change in nomenclature of gamete donation from “anonymous” to “nonidentified” to better reflect the reality that true anonymity no longer exists.

**Clinical question**

What constitutes best practices in disclosure to offspring of the fact that they are donor-conceived? When is the optimal time to disclose? Are there risks that accompany nondisclosure?

## Expert approach

Gamete donation provides a safe and effective method of family building for many individuals and couples who would otherwise be unable to become parents. At its inception, the practice was shrouded in secrecy and performed without consent. In 1884, a young couple struggling with infertility consulted Dr. Pancoast at Jefferson Medical College. The husband was diagnosed with azoospermia, likely due to a prior infection. During the procedure, and without the wife's knowledge or explicit consent, she was anesthetized with chloroform and “some fresh semen from the best-looking member of the class was deposited in the uterus (1).” The resulting pregnancy was successful, and the couple had a son. Initially, neither parent was informed about the nature of the procedure. Eventually, the physician disclosed the details to the husband, who chose not to share the information with his wife. It remains unclear whether the child ever learned of his donor conception.

Over the intervening decades, support for secrecy continued to dominate discussions of third-party reproduction, including within the medical community. Over 60 years ago, *Fertility and Sterility* published an article stating that couples conceiving via donor sperm “should be advised for their own protection that discussion of this procedure should preferably be limited to their physician and themselves, or at most to include their religious counselor. It is also the physician's duty to maintain the same degree of secrecy of records and anonymity between donors and recipients (2).”

More recently, thanks in part to the availability of direct-to-consumer DNA testing, social media, and the internet, donor-conceived people have been able to learn their genetic origins and, in many cases, identify their gamete donor(s). The unveiling of this shroud of secrecy led to a questioning of the long-held belief that anonymity should be maintained at all costs. Common wisdom had previously dictated that both gamete donors and intended parents favored anonymity. Although this might hold some truth, one critical group of stakeholders has long been generally left out of the conversation. The voices of the donor-conceived people (DCP), which had previously been silenced in favor of “protecting” the gamete donors and intended parents, finally began to be heard.

It is estimated that >1 million DCP are currently living in the United States (3). Many of these DCP are now adults, and it is unknown what percentage of them are aware that they are donor-conceived. Heterosexual couples initially accounted for the majority of donor sperm cycles in the early years of this practice, but the introduction of intracytoplasmic sperm injection obviated the need for donor sperm in many couples whose infertility was due to male factor (4). At present, it appears that 50% of donor sperm is utilized by lesbian couples, and 25% by single females, representing exponential growth in these populations (5). This changing demographic has shifted attitudes toward donor sperm use, given that its use in these populations is largely undeniable.

Given that DCP are highly likely to eventually unearth the circumstances of their conception, it is critical to optimize the timing and method by which such disclosure occurs. A growing body of evidence points in favor of early and intentional disclosure, making it part of their origin story and including more details as the child’s developmental stage dictates. Optimally, children should grow up feeling that they have always known that they were donor-conceived. One study found that children whose parents shared this information with them before they reached 7 years of age exhibited more positive psychological adjustment and family relationships than those who learned it at later ages (6). Additional research indicates that early disclosure fosters greater trust between children and their parents and is associated with stronger, more secure family relationships (7, 8).

Physicians are often the first professionals to counsel individuals or couples pursuing family building with gamete donation, placing them in a unique position to lay the foundation for future DCP even before conception occurs. Physicians should be equipped with appropriate resources to support these patients, including educational materials on disclosure practices and referrals to mental health professionals with expertise in third-party reproduction. Early in the process, physicians should emphasize the importance of transparent communication with future children and offer guidance on how and when to disclose donor conception. Since 2004, the American Society for Reproductive Medicine (ASRM) Ethics Committee has recommended that DCP be informed of their origins, and current ASRM Practice Committee guidelines advocate for psychosocial counseling for all patients considering donor gametes (9, 10). Initiating these conversations from the outset promotes secure parent-child relationships and helps prevent the harm associated with unintentional or late disclosure, both of which have been shown to negatively impact family dynamics (11, 12).

All this said, when navigating the complexities of disclosure, it is important to distinguish between informing a child that they are donor-conceived and revealing the identity of the gamete donor. Although many gamete banks permit identity disclosure once the donor-conceived individual reaches the age of 18, earlier access to this information may occur—for instance, if an older donor-conceived sibling has already obtained the donor’s identity. However, disclosing donor identity before the legal age of majority can pose risks to family stability, particularly in LGBTQ+ families living in states where parental rights lack comprehensive legal protection. In such cases, families considering early identity disclosure are strongly encouraged to seek legal counsel to understand potential implications and safeguards.

Ethically, withholding the fact that a child is donor-conceived undermines their right to personal autonomy. Without knowledge of their genetic origins, an individual’s capacity for self-determination is significantly limited. In alignment with the principle of nonmaleficence, parents who conceive through gamete donation should be encouraged to share this information with their children early and often. Early, age-appropriate disclosure helps prevent the potential psychological harm and trust issues that can arise when individuals discover their donor-conceived status later in life.

## Learning points

• Before initiating third-party reproduction, intended parents should understand how, when, and under what circumstances information about donors will be provided to them.
• Counseling for intended parents should focus on the long-term implications of becoming parents via donor conception with the goal of increasing their confidence in the process and promoting the health and well-being of the DCP and their families. Parents may benefit from ongoing counseling over time to assist them in navigating issues of disclosure and potential relationships between DCP and their gamete donors.
• Given the availability of direct-to-consumer genetic testing, social media, and the internet, parents no longer have a choice as to *whether* their donor-conceived child will eventually learn they are donor-conceived, but they do have a choice as to *how* their child will learn this information.
• Early disclosure is associated with more positive outcomes and prevents the potential negative effects of accidental disclosure and late discovery in adulthood.
• Continuing education and training of medical, mental health, and other allied professionals (including those not specializing in this area) should reflect the evolving needs and interests of DCP and their families. This training should avoid propagation of outdated concepts (e.g., allowing an assumption that the donation is “closed” or “anonymous”).

## Conclusion

The community of DCP is broad and varied and continues to grow. It is the responsibility of the physician to ensure that the children who will ultimately result from gamete and embryo donation receive the respect and the consideration that they deserve as their parents navigate the best ways to convey to them the circumstances of their conception. This includes early and ongoing discussions between parent and child about the fact that they are donor-conceived. In such a way, families can maximize the chance that there will be strong, supportive, and lasting relationships between parents and their donor-conceived children.

## CRediT Authorship Contribution Statement

**Sigal Klipstein:** Writing – original draft. **Lauri A. Pasch:** Writing – original draft.

## Declaration of Interests

S.K. has nothing to disclose. L.A.P. has nothing to disclose.

## References

[1] Hard A.D. (1909). Artificial impregnation. Med World.

[2] Behrman S.J. (1959). Artificial insemination. Fertil Steril.

[3] Arocho R., Lozano E.B., Halpern C.T. (2019). Estimates of donated sperm use in the United States: National survey of family growth 1995–2017. Fertil Steril.

[4] Shapiro S., Saphire D.G., Stone W.H. (1990). Changes in American A.I.D. practice during the past decade. Int J Fertil.

[5] Diego D., Medline A., Shandley L.M., Kawwass J.F., Hipp H.S. (2022). Donor sperm recipients: fertility treatments, trends, and pregnancy outcomes. J Assist Reprod Genet.

[6] Golombok S., Jones C., Hall P., Foley S., Imrie S., Jadva V. (2023). A longitudinal study of families formed through third-party assisted reproduction: mother–child relationships and child adjustment from infancy to adulthood. Dev Psychol.

[7] Jadva V., Freeman T., Kramer W., Golombok S. (2009). The experiences of adolescents and adults conceived by sperm donation: comparisons by age of disclosure and family type. Hum Reprod.

[8] Scheib J.E., Riordan M., Rubin S. (2005). Adolescents with open-identity sperm donors: reports from 12–17 year olds. Hum Reprod.

[9] The Practice Committee of the American Society for (2024). Reproductive Medicine and the Practice Committee for the Society for Assisted Reproductive Technology. Gamete and embryo donation guidance. Fertil Steril.

[10] Ethics Committee of the American Society for Reproductive Medicine (2019). Interests, obligations, and rights in gamete and embryo donation: an Ethics Committee opinion. Fertil Steril.

[11] Widbom A., Sydsjö G., Lampic C. (2022). Psychological adjustment in disclosing and non-disclosing heterosexual-couple families following conception with oocytes or spermatozoa from identity Elease donors. Reprod Biomed Online.

[12] Pasch L.A. (2018). New realities for the practice of egg donation: a family-building perspective. Fertil Steril.

